# Supplementary material for: Deletion of FNDC5/Irisin modifies murine osteocyte function in a sex-specific manner
Source: bioRxiv. 2023 Nov 6:2023.11.06.565774. Preprint. [Version 1] doi: 10.1101/2023.11.06.565774 (PMC10659274; doi:10.1101/2023.11.06.565774)
Supplement: 1 [file NIHPP2023.11.06.565774V1-supplement-1.pdf]

bioRxiv preprint doi: https://doi.org/10.1101/2023.11.06.565774; this version posted November 6, 2023. The copyright holder for this preprint (which was not certified by peer review) is the author/funder, who has granted bioRxiv a license to display the preprint in perpetuity. It is made available under aCC-BY 4.0 International license.

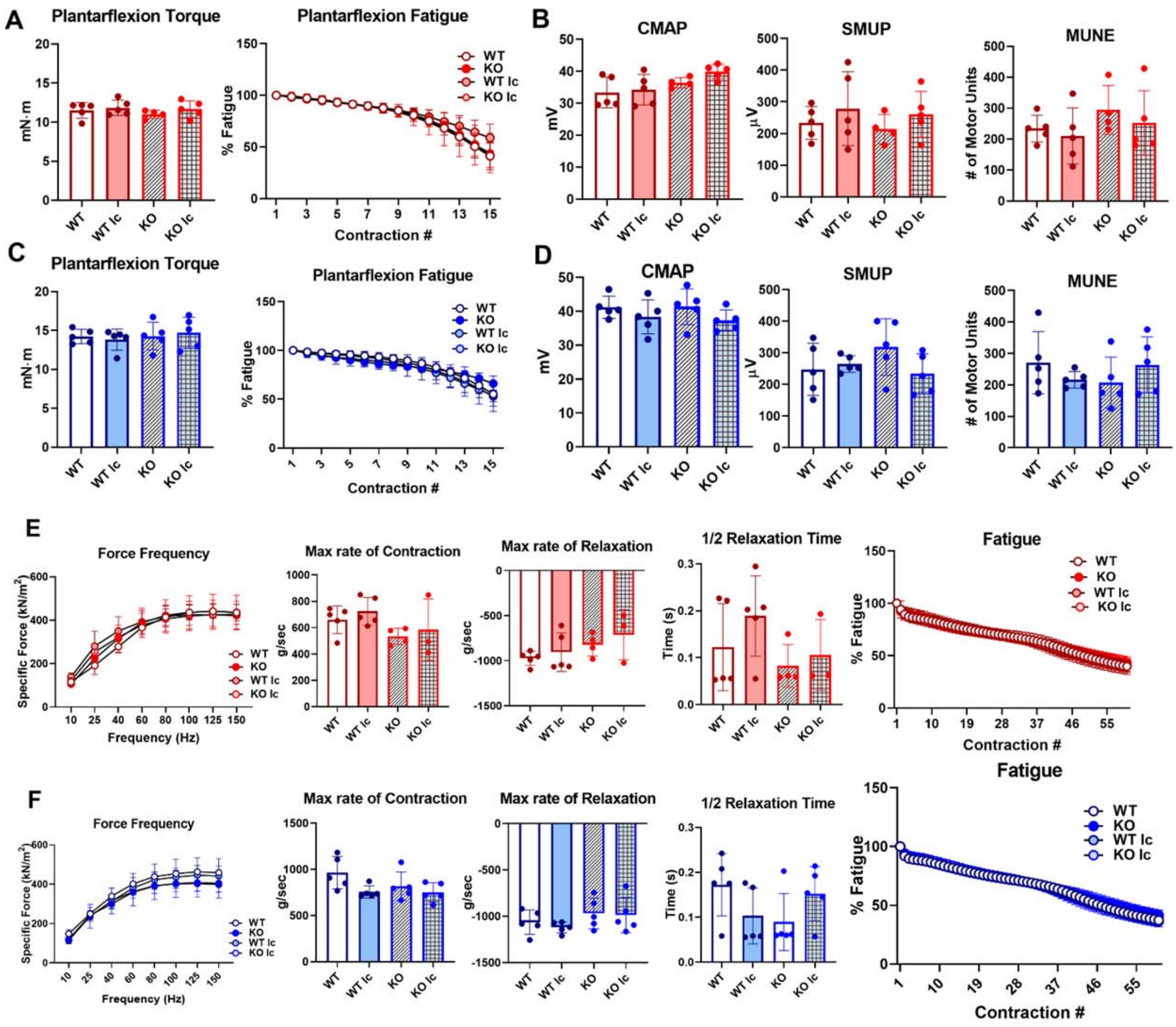

Supplementary Figure 1: Neither genotype nor dietary calcium alters muscle functions *in vivo* or *ex vivo*

Panels A and C show *in vivo* muscle plantarflexion force (reported as plantarflexion torque and plantarflexion fatigue) in WT and KO female (A) and male (C) mice on a control or a low calcium diet, panels B and D show muscle electrophysiology parameters of CMAP, SMUP, and MUNE in WT and KO female (B) and male (D) mice, and panels E and F show *ex vivo* EDL functional measurement (reported as specific force frequency, maximum rate of contraction, maximum rate of relaxation, half-relaxation time, and % fatigue) in WT and KO female (E) and male (F) mice

2-way ANOVA with Tukey's post hoc test was done. n= 4-5/group. As depicted here, red is female,

bioRxiv preprint doi: https://doi.org/10.1101/2023.11.06.565774; this version posted November 6, 2023. The copyright holder for this preprint (which was not certified by peer review) is the author/funder, who has granted bioRxiv a license to display the preprint in perpetuity. It is made available under aCC-BY 4.0 International license.

and blue is male.

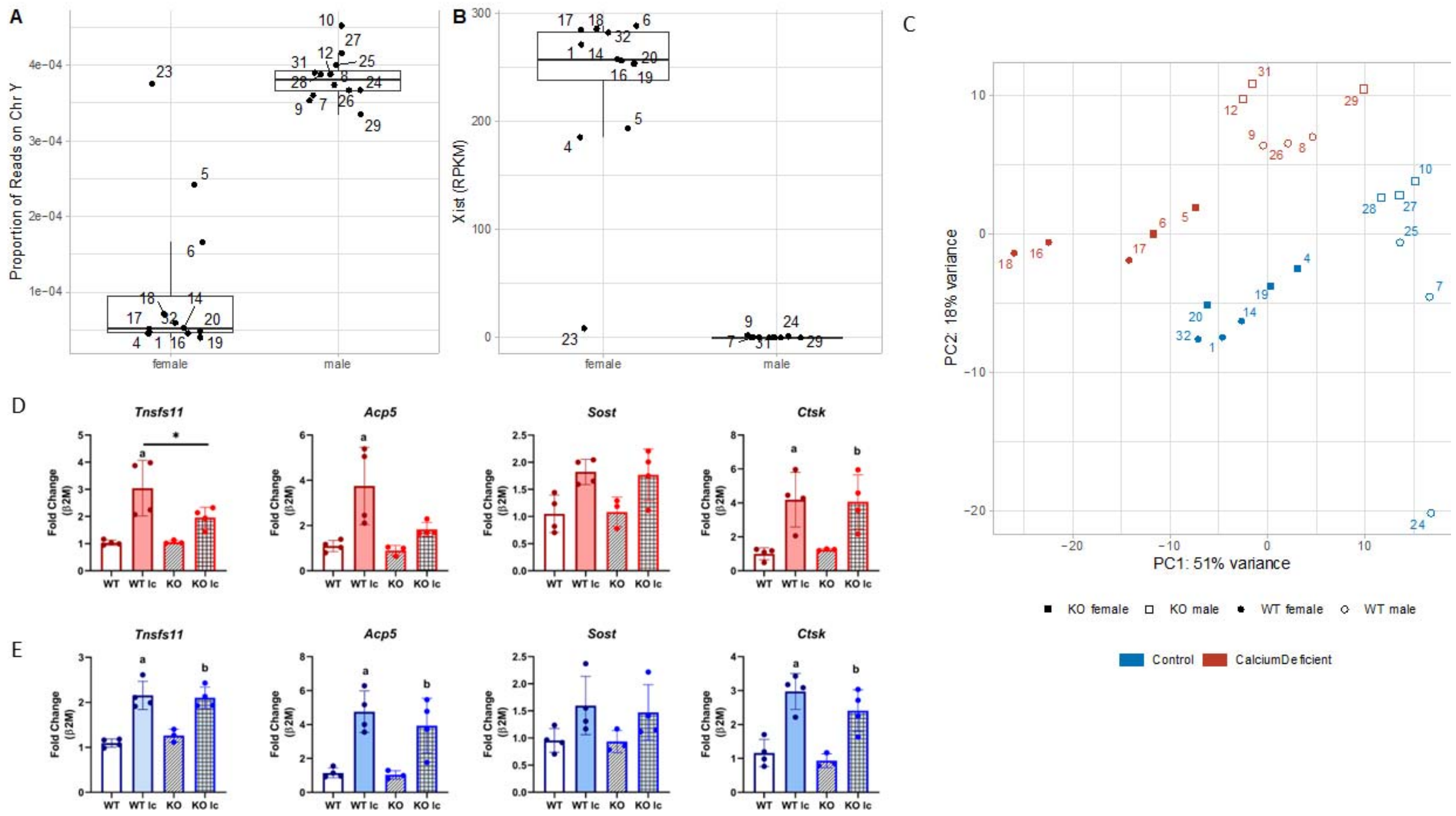

Supplementary Figure 2: Quality control and validation of RNA sequencing

Sanity check of data on the sample's sex. **A:** Boxplot of proportional of reads on chromosome Y. Male should have a higher value than female. **B**: Boxplot of RPKM of Xist. Males should have very low expression of Xist. **C**: Scatter plot of PC1 and PC2 from Principal Component Analysis (PCA) of gene expression data. **D**: qPCR analysis of Tnsfs11, Acp5, Sost, and Ctsk genes from osteocyte-enriched bone chips from female samples. n= 3-4/sample. Two-way ANOVA with Tukey's post-hoc test was performed for statistical analysis. Gene fold-change was normalized using β-2-microglobulin as the housekeeping gene. a= Significantly different from WT, b= Significantly different from KO, *= $p < 0.05$. **E**: qPCR analysis of Tnsfs11, Acp5, Sost, and Ctsk genes from osteocyte-enriched bone chips from male samples. n= 3-4/sample. Two-way ANOVA with Tukey's post-hoc test was performed for statistical analysis. Gene fold-change was normalized using β-2-microglobulin as the housekeeping gene. a= Significantly different from WT, b= Significantly different from KO, *= $p < 0.05$.

bioRxiv preprint doi: https://doi.org/10.1101/2023.11.06.565774; this version posted November 6, 2023. The copyright holder for this preprint (which was not certified by peer review) is the author/funder, who has granted bioRxiv a license to display the preprint in perpetuity. It is made available under aCC-BY 4.0 International license.

Bone Parameters	Virgin		Lactation	
	WT	KO	WT	KO
**Femoral cortical bone parameters**				
Ct. B. Ar/T. Ar (%)	47.4 ± 1.2	48 ± 1	35.2 ± 1.8[a]	37.5 ± 1.8[b, c]
Ct. Th (mm)	0.18 ± 0.004	0.19 ± 0.005	0.13 ± 0.004[a]	0.14 ± 0.01[b, c]
Ps. Pm (mm)	5.16 ± 0.2	5.2 ± 0.06	5.18 ± 0.16	5.2 ± 0.14
Es. Pm (mm)	3.95 ± 0.1	4 ± 0.13	4.4 ± 0.11[a]	4.3 ± 0.09[b]
Marrow cavity area (mm^2)	0.93 ± 0.1	0.93 ± 0.04	1.16 ± 0.05[a]	1.13 ± 0.05[b]
**Femoral trabecular bone parameters**				
BV/TV (%)	3.7 ± 1	4.5 ± 0.8	3.1 ± 0.7	4 ± 1.1
Tb. Th (mm)	0.043 ± 0.002	0.044 ± 0.001	0.039 ± 0.002[a]	0.039 ± 0.001[b]
Tb. Sp (mm)	0.37 ± 0.05	0.36 ± 0.03	0.57 ± 0.15[a]	0.44 ± 0.09
Tb. N (1/mm)	0.85 ± 0.2	1.06 ± 0.2	0.8 ±0.2	1.04 ± 0.25

Bone parameters	Change	% Change	
		WT	KO
Cortical Bone Area Fraction	Decrease	26%	22% *
Cortical Thickness	Decrease	29%	24% *
Ultimate Force	Decrease	38%	31% *
Osteoclast Number/ bone parameter	Increase	141%	129%
TRAP-positive osteocytes	Increase	101%	175% *
Lacunar Area	Increase	26%	15% *
Serum RANKL	Increase	170%	80% *

Supplementary Table 1: FNDC5 KO mice femurs are partially resistant to lactation-induced bone loss.

Femoral cortical and trabecular bone parameters of WT and FNDC5 KO female virgin and

bioRxiv preprint doi: https://doi.org/10.1101/2023.11.06.565774; this version posted November 6, 2023. The copyright holder for this preprint (which was not certified by peer review) is the author/funder, who has granted bioRxiv a license to display the preprint in perpetuity. It is made available under aCC-BY 4.0 International license.

lactation mice. n = 5-8/group. a= significant compared to WT control, b= significant compared to KO control, c= significant compared to WT low Ca diet, 2-way ANOVA with Tukey's post hoc test, significance <0.05, n= 8/group. Percentage change in different bone and serum parameters in WT and FNDC5 KO female mice with lactation. *= p<0.05 compared to WT.

Bone Parameters	Female Normal Diet		Female Low Ca Diet		Male Normal Diet		Male Low Ca Diet	
	WT	KO	WT	KO	WT	KO	WT	KO
***Ex vivo* femur DXA**								
BMD (mg/cm^2)	75.4± 2.4	76.6± 1.5	65.4± 4.3[a]	71.4± 3.4 [c]	74.6± 1.5	78.3± 3[a]	68.2± 3	68.1± 2[b]
BMC (g)	0.03± 0.002	0.03± 0.001	0.024± 0.002[a]	0.027± 0.002 [b,c]	0.029± 0.002	0.032± 0.004	0.026± 0.002	0.025± 0.003[b]
**Femoral cortical bone parameters**								
Ct. B.Ar/T.Ar%	47.8± 1.6	48.4± 0.4	41.6± 1.1[a]	45.2± 1.4[b, c]	40.1± 1.4	43.6± 0.6[a]	38.3± 0.9	39.1± 1.2[b]
Ct. Th (mm)	0.2± 0.01	0.2± 0.01	0.15± 0.01[a]	0.17± 0.01[b, c]	0.15± 0.01	0.2± 0.01[a]	0.14± 0.01	0.14± 0.01[b]
Marrow Cavity Area	0.92 ± 0.04	0.86± 0.02	1.02 ± 0.06[a]	0.9 ± 0.02[c]	1.1 ± 0.04	1.03 ± 0.06[a]	1.2 ± 0.03	1.08 ± 0.03[c]
**Femoral trabecular bone parameters**								
BV/TV (%)	3.6 ± 1.2	4.3 ± 1	3.2 ± 1	3.9 ± 1	6.1 ± 1.1	8.7 ± 1.9	5.3 ± 1.2	6.4 ± 0.6
Tb. Th (mm)	0.059± 0.002	0.059 ± 0.004	0.056± 0.002	0.055± 0.001	0.036 ± 0.001	0.035 ± 0.001	0.035±0 .001	0.035 ± 0.002
Tb. Sp (mm)	0.38 ± 0.03	0.35 ± 0.02	0.51 ± 0.12[a]	0.48 ± 0.08[b]	0.274 ± 0.025	0.235 ± 0.021	0.278 ± 0.027	0.265 ± 0.01
Tb. N (1/mm)	0.81 ± 0.2	0.95 ± 0.14	0.7 ± 0.02	0.91 ±0.13	1.7 ± 0.34	2.5[a] ± 0.5	1.5 ± 0.3	1.8 ± 0.1
**Femoral mechanical properties**								
Ultimate	19±	19.4±	14.8±	16.4±	18.3±	17.6±	15±	12.7±

bioRxiv preprint doi: https://doi.org/10.1101/2023.11.06.565774; this version posted November 6, 2023. The copyright holder for this preprint (which was not certified by peer review) is the author/funder, who has granted bioRxiv a license to display the preprint in perpetuity. It is made available under aCC-BY 4.0 International license.

Force (N)	1	1.15	0.7[a]	0.5[b]	1	0.9	1.3[a]	1.5[b, c]
Stiffness (N/mm)	78.6± 3.2	79.1± 4.9	56.8± 5[a]	67± 4.3	76.7± 5.6	56.4± 4.75[a]	56± 10.2[a]	48.5± 4.9[b, c]
Energy to Failure (N)	2.9± 0.3	3.1± 0.6	1.8± 0.5[a]	2± 0.3[b]	3.6± 0.9	3.01± 0.6	2.5± 0.3[a]	2.35± 0.14

Supplementary Table 2: WT and FNDC5 KO female and male mice bone responds differently to a low-calcium diet

Femoral BMD, BMC, cortical and trabecular bone parameters, and mechanical properties of 4-5-month-old WT and KO female and male mice under a normal diet or a 2-week low calcium diet. n = 5/group. a= significant compared to WT control, b= significant compared to KO control, c= significant compared to WT low Ca diet, 2-way ANOVA with Tukey's posthoc test, significance <0.05, n= 4-5/group.
